# Selective Targeting of TNF Receptors as a Novel Therapeutic Approach

**DOI:** 10.3389/fcell.2020.00401

**Published:** 2020-05-26

**Authors:** Roman Fischer, Roland E. Kontermann, Klaus Pfizenmaier

**Affiliations:** Institute of Cell Biology and Immunology, University of Stuttgart, Stuttgart, Germany

**Keywords:** TNF, TNFR1, TNFR2, therapy, inflammation, tissue regeneration

## Abstract

Tumor necrosis factor (TNF) is a central regulator of immunity. Due to its dominant pro-inflammatory effects, drugs that neutralize TNF were developed and are clinically used to treat inflammatory and autoimmune diseases, such as rheumatoid arthritis, inflammatory bowel disease and psoriasis. However, despite their clinical success the use of anti-TNF drugs is limited, in part due to unwanted, severe side effects and in some diseases its use even is contraindicative. With gaining knowledge about the signaling mechanisms of TNF and the differential role of the two TNF receptors (TNFR), alternative therapeutic concepts based on receptor selective intervention have led to the development of novel protein therapeutics targeting TNFR1 with antagonists and TNFR2 with agonists. These antibodies and bio-engineered ligands are currently in preclinical and early clinical stages of development. Preclinical data obtained in different disease models show that selective targeting of TNFRs has therapeutic potential and may be superior to global TNF blockade in several disease indications.

## Introduction

Tumor necrosis factor (TNF) is a key regulatory component of the immune system that regulates innate and adaptive immunity and contributes to initiation and maintenance of inflammation (Aggarwal, 2003). The major cellular source of TNF are macrophages and immune cells that are activated in response to infections or tissue damage (Fischer and Maier, 2015). Therefore, regulated TNF expression is essential to promote tissue homeostasis and fight infections. In contrast, deregulated TNF expression and signaling may induce pathology resulting in chronic inflammation and tissue damage. Indeed, increased levels of TNF were identified in patients with autoimmune and degenerative diseases (Fischer and Maier, 2015; Monaco et al., 2015). To counteract the pro-inflammatory and tissue degenerative effects of TNF signaling, therapeutics have been developed that neutralize TNF. Currently, five structurally different anti-TNF drugs are approved for clinical use: infliximab (Remicade), adalimumab (Humira), certolizumab pegol (Cimzia), golimumab (Simponi), and etanercept (Enbrel) (Monaco et al., 2015). These anti-TNF therapeutics, and biosimilars of infliximab, etanercept and adalimumab that have been approved recently, are successfully used to treat autoimmune diseases, including RA, juvenile RA (JRA), IBD, psoriasis, and ankylosing spondylitis (AS) (Monaco et al., 2015). Despite the clinical success of anti-TNF therapeutics they also show limitations, such as their restricted responsiveness, and severe side-effects, such as opportunistic infections, invasive fungal infections, reactivation of tuberculosis, and development of other autoimmune diseases and lymphomas (Tracey et al., 2008; Monaco et al., 2015). Further, clinical evaluation of anti-TNF therapy in multiple sclerosis failed (van Oosten et al., 1996; Lenercept Study Group, 1999) and anti-TNF therapy of juvenile rheumatoid arthritis resulted in development of MS-like exacerbations and demyelinating lesions in some patients (Sicotte and Voskuhl, 2001). Altogether this indicates that the use of anti-TNF drugs is limited and contraindicative for several indications, including neurodegenerative diseases.

The limitations of anti-TNF therapy may depend on TNF’s pleiotropic biological functions via two distinct TNF receptors (TNFR). Synthesized as a transmembrane protein (tmTNF), the tmTNF form can activate both, TNFR1 and TNFR2. After proteolytical processing, the soluble trimers (sTNF) mainly activate TNFR1 (Fischer et al., 2015). In different animal disease models, genetic deletion of TNFR1 is typically associated with lack or reduced disease, whereas TNFR2 ablation exacerbates disease. These and other data indicate that sTNF/TNFR1 signaling mainly mediates pro-apoptotic and inflammatory responses, whereas TNFR2 contributes to immune regulation and tissue regeneration. Therefore, reagents that selectively target TNFRs might be superior to global TNF blockade because they allow a differential activation and/or inhibition of TNFRs.

Lymphotoxin-α (LTα) is another homotrimeric ligand of the TNF superfamily (TNFSF) that shares 50% homology with TNF (Gray et al., 1984) and can also bind to TNFR1 and TNFR2 (Bodmer et al., 2002). In contrast to TNF, LTα lacks the transmembrane domain and is therefore only expressed as a soluble homotrimeric form (Ruddle, 2014). The close tertiary and quaternary structures indicate that TNF and LTα are functionally redundant. However, the involvement of LTα in inflammatory diseases is less well characterized than sTNF and a RA clinical trial using the anti-lymphotoxin-alpha antibody pateclizumab did not show statistically significant improvement in RA signs and symptoms (Kennedy et al., 2014). Differences between sTNF and LTα have been described elsewhere (Ruddle, 2014; Hirose et al., 2018). In this review, we will summarize the current knowledge of signal pathways emanating from the two TNFRs, their patho-/physiologic role and discuss recent promising results obtained in different disease models in the pre-clinical development of novel TNFR selective drugs.

## Tumor Necrosis Factor

Tumor necrosis factor is synthesized as a 26 kDa type II transmembrane protein that assembles into a homotrimeric molecule (tmTNF) (Kriegler et al., 1988) that can be proteolytically cleavage by the matrix metalloproteases (MMP) TNFα-converting enzyme (TACE/ADAM17) resulting in soluble TNF homotrimers (sTNF; 51 kDa) (Black et al., 1997). TNF binds to the two type I transmembrane receptors TNFR1 and TNFR2. Both TNF receptors contain four cysteine-rich domains (CRD) in their extracellular domains. The membrane distal CRD contains the preligand binding assembly domain (PLAD), which is important for ligand-mediated formation of active receptor complexes. In the absence of a ligand, the PLAD mediates inactive self-association of homo-multimerized receptors (Chan et al., 2000). TNFR1 is constitutively expressed on almost all nucleated cells. In contrast, the expression of TNFR2 is more restricted, highly regulated on various cells of the immune system, and plays an important role, too, on cells of the vasculature, muscle and brain tissues (Wajant et al., 2003; Fischer and Maier, 2015; Pegoretti et al., 2018).

Interestingly, sTNF and tmTNF have different activities to stimulate signaling via TNFR1 and TNFR2. Despite binding sTNF with subnanomolar affinity, TNFR2 needs tmTNF for robust activation (Grell et al., 1995). This difference might be due to different association/dissociation kinetics of the TNF/TNFR complexes. TNF binds to TNFR1 with a higher affinity (K_d_ = 1.9 × 10^–11^ M) than TNFR2 (K_d_ = 4.2 × 10^–10^ M) (Grell et al., 1998). This high affinity for TNFR1 is dependent on stabilization of the TNF/TNFR1 complex, whereas short-lived signaling-incompetent complexes are formed by transient binding of sTNF to TNFR2 (Grell et al., 1998; Krippner-Heidenreich et al., 2002). Stoichiometry analysis revealed differences in ligand/receptor interactions between TNFR1 and TNFR2 and indicated that avidity is an important factor for TNF-binding and downstream signaling of TNFR2 (Boschert et al., 2010). Indeed, using a system with ligand-immobilization on a surface in a nanoscaled pattern with defined spacings, Ranzinger et al. (2009) showed that mere mechanical fixation of TNF was sufficient to activate TNFR1 but not TNFR2. Whereas, robust TNFR2 activation was dependent on additional stabilization by cluster formation (Ranzinger et al., 2009). Altogether, these data clearly indicate that tmTNF-mediated cluster formation of tmTNF/TNFR2 complexes is necessary for robust activation of TNFR2.

The membrane-proximal extracellular stalk regions were identified as a crucial determinant in controlling responsiveness to sTNF (Richter et al., 2012). Richter et al. (2012) showed that the arrangement of the TNFRs in the plasma membrane in the absence of ligand is a fundamental parameter determining the responsiveness of TNFRs to sTNF. Indeed, the stalk region of TNFR2, in contrast to the corresponding part of TNFR1, efficiently inhibited clustering of TNFR2 in particular cell membrane regions and ligand-independent PLAD-mediated homotypic receptor preassembly resulting in abolished sTNF-, but not tmTNF-induced signaling (Richter et al., 2012). These data are supported by a report suggesting that the two TNFRs are topological segregated in different plasma membrane microcompartments independent of the cytoplasmic signaling domains of the receptors (Gerken et al., 2010). The intracellular structure of the TNFRs is highly different and defines their activity. TNFR1 belongs to the family of death domain (DD)-containing receptors, whereas TNFR2 is a TRAF-interacting receptor without DD (Wajant et al., 2003).

## TNFR Signaling

### TNFR1

**FIGURE 1:**
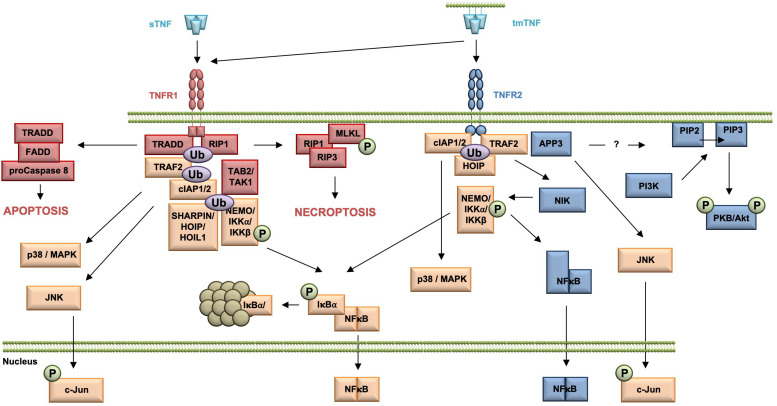
Overview of the TNFR1 and TNFR2 signaling pathway. All TNFR1-exclusive signaling mediators are marked red, whereas all TNFR2-exclusive signaling components are shown in blue. All mediators used by both pathways are labeled orange.

Upon TNF binding, TNF receptor 1 associated protein with death domain (TRADD), the receptor interacting protein kinase 1 (RIP1), TNF receptor associated factor 2 (TRAF2), and the cellular inhibitor of apoptosis proteins (cIAPs) 1 and 2 are recruited to the receptor (Figure 1). The cIAPs modify intracellular binding partners of the TNFR1 signaling complex (TNFR1-SC), in particular RIPK1, with K63-linked ubiquitin chains to create a docking platform for the linear ubiquitin assembly complex (LUBAC). LUBAC then adds linearly linked ubiquitin chains to RIPK1 leading to the recruitment of the inhibitor of kappa B kinases (IKK) complex and the MAP3K transforming growth factor-ß (TGFß)–activated kinase-1 (TAK1), which binds to the TNFR1 complex via the adapter protein TAK1-binding protein-2 (TAB2). TAK1 phosphorylates IKKβ and LUBAC adds linear ubiquitin to NEMO, both components of the IKK complex. IKK then phosphorylates inhibitor of kappa B-alpha (IκBα) leading to its ubiquitination and subsequent proteasomal degradation. The dissociation of IκB from the transcription factor nuclear factor kappa B (NFκB) releases its nuclear localization sequence (NLS) resulting in the nuclear translocation of free NFκB dimers and transcription of NFκB-regulated targets (Wajant and Scheurich, 2011; Schmukle and Walczak, 2012). Next to the classical NFκB pathway, the TNFR1 signaling complex I can bind and activate distinct MAP kinase kinases (MKK) resulting in the activation of p38 MAP kinase and JNK pathway (Natoli et al., 1997; Brinkman et al., 1999). The signaling complex I can be internalized, which leads to the dissociation of TRAF2 and the cIAPs and the subsequent recruitment of the adaptor protein Fas associated death domain protein (FADD) and the procaspase 8 to form the secondary pro-apoptotic signaling complex II. Within the death inducing signaling complex (DISC), procaspase 8 is activated by autocatalytic cleavage resulting in activation of the effector caspase cascade ultimately leading to induction of apoptosis (Micheau and Tschopp, 2003; Schneider-Brachert et al., 2004). Using a systems biology approach and mathematical modeling temporal responses of TNFR1-mediated cell death induction were described. A global sensitivity analysis uncovered that concentrations of Caspase-8 and Caspase-3, and their respective inhibitors FLIP, BAR, and XIAP are key elements for deciding the cell’s fate. In contrast, NFκB-mediated anti-apoptotic signaling pathways delayed the time of death (Schliemann et al., 2011). When caspase 8 is absent or inactivated, kinase-active RIPK1 recruits and activates RIPK3, resulting in the formation of the necrosome. As a constitutive binding partner of RIPK3, mixed lineage kinase domain-like protein (MLKL) is incorporated in the necrosome (Grootjans et al., 2017). Phosphorylation of MLKL results in a conformational change, recruitment to the plasma membrane and execution of necroptosis via membrane permeabilization (Vanden Berghe et al., 2014; Grootjans et al., 2017).

### TNFR2

In contrast to the very well characterized TNFR1 signaling pathways and their physiologic relevance early in TNF research, TNFR2-mediated signaling pathways and in particular their role in TNF biology were uncovered much later (Figure 1). TNFR2 activation results in recruitment of TRAF2 (Rothe et al., 1994), cIAP1/cIAP2 (Rothe et al., 1995a), and HOIP, a LUBAC component (Borghi et al., 2018), which form the TNFR2 signaling complex (SC). cIAP-mediated K63-linked polyubiquitination of the SC is required for recruitment of HOIP, which mediates M1-ubiquitination (Borghi et al., 2018). Both HOIP and cIAP1 are required for TNFR2-induced canonical NFκB activation via IKKβ (Rothe et al., 1995b; Borghi et al., 2018). In addition, in contrast to TNFR1, TNFR2 was shown to be capable to induce the non-canonical NFκB pathway (Rauert et al., 2010). After degradation of TRAF2, probably through receptor internalization and lysosomal degradation (Fischer et al., 2011a), the kinase NIK accumulates, phosphorylates and activates IKKα. This leads to processing of the p100 subunit of NFκB to p52 and the subsequent nuclear translocation of p52/RelB NFκB heterodimers (Sun, 2017).

Similar to TNFR1 and TNFR2 activation may result in induction of the c-Jun N-terminal kinase (JNK) (Jupp et al., 2001) and the p38 MAPK pathway (Inoue et al., 2015; He et al., 2018). Interestingly, recently mitochondrial aminopeptidase P3 (APP3, also known as XPNPEP3) was identified as a novel component of the TNFR2 signal complex, which regulates TNF–TNFR2-dependent phosphorylation of JNK (Inoue et al., 2015). The authors describe that APP3 is released from mitochondria in a TNF-defendant way in the absence of mitochondrial outer membrane permeabilization (MOMP) and suggest that APP3 exerts an anti-apoptotic function (Inoue et al., 2015). Interestingly, it was shown that TNFR2 ligation enhances cell proliferation through the non-canonical NFκB pathway in human regulatory T cells (Tregs) (Wang et al., 2018), whereas in mouse Tregs activation of p38 MAPK was important for TNFR2-induced proliferation (He et al., 2018). Furthermore, TNFR2 promotes phosphatidylinositol 3-kinase (PI3K)-dependent phosphorylation of the protein kinase PKB/Akt via a yet unknown mechanism (Marchetti et al., 2004; Fischer et al., 2011b). Here, PI3K phosphorylates the D3 hydroxyl group of the inositol ring of the plasma membrane lipid phosphatidylinositol-4,5-bisphosphate (PIP2) resulting in the second messenger phosphatidylinositol 3,4,5-bisphosphate (PIP3) (Cantley, 2002). PKB/Akt then is recruited to the plasma membrane by direct binding to PIP3 through its pleckstrin-homology (PH) domains (Lawlor and Alessi, 2001). There, PKB/Akt undergoes a conformational change and is phosphorylated at residue threonine 308 in the activation loop (T loop) of the kinase domain by PDK-1 (Alessi, 2001) and at residue serine 473 in the hydrophobic motif by the Rictor/mammalian target of rapamycin (mTOR) complex (Sarbassov et al., 2005). Activated PKB/Akt then promotes cell survival and proliferation (Fischer et al., 2015; Ortí-Casañ et al., 2019).

## Opposing Roles of TNFR1 and TNFR2

### Inflammatory Diseases

Tumor necrosis factor plays an important role for regulation of the adaptive and innate immune system and thus, is a key player for both infectious and non-infectious inflammatory disorders. Interestingly, TNF induces opposing effects in the immune system, i.e., it plays a key role for the initiation and orchestration of inflammation, while it also suppresses immune cell activity. These antithetic effects often can be explained by the diverse signaling mediated via TNFR1 and TNFR2 (Figure 1).

TNFR1 is expressed on a multitude of effector immune cells and most described TNF-mediated proinflammatory functions are predominantly mediated via TNFR1 (Fischer and Maier, 2015; Fischer et al., 2015; Mehta et al., 2018). In contrast, TNFR2 expression is more restricted and highly regulated. In immunity, TNFR2 expression is predominantly found on activated T cells and, in particular, is critically involved in regulation of immune responses through signaling in regulatory T cells (Tregs), a specific immune modulatory lymphocyte subpopulation that suppress development of autoimmune diseases. In particular, it was shown that the expression level of TNFR2 is correlated to the suppressive potential of natural Tregs (nTregs) (Chen et al., 2007, 2008, 2010b), indicating that the most potent suppressors are highly susceptible to TNFR2 activation. It is well recognized now that TNFR2 contributes to the expansion of CD4^+^FoxP3^+^ nTregs *in vitro* and *in vivo* (Chen et al., 2007, 2008; Okubo et al., 2013; Chopra et al., 2016; Fischer et al., 2017, 2018, 2019a,b; Padutsch et al., 2019) and the stabilization of the CD4^+^Foxp3^+^ Treg phenotype in the inflammatory environment (Chen et al., 2013). Like CD4^+^ Tregs, CD8^+^ suppressor cells can express FoxP3 and CD25. Similar to CD4^+^ Tregs, the most potent CD8^+^ suppressors are characterized by the expression of TNFR2 (Ablamunits et al., 2010; Horwitz et al., 2013).

#### Infectious Diseases

TNFR1 plays an essential role for host defense against various pathogenic organisms. Rothe et al. described that TNFR1^–/–^ mice were resistant to TNF-mediated toxicity [low-dose lipopolysaccharide (LPS) after sensitization with D-galactosamine (D-GalN)], whereas they are still sensitive to elevated doses of LPS only treatment (Rothe et al., 1993). In addition, they are highly susceptible to infection with the facultative intracellular bacterium *Listeria monocytogenes* (Rothe et al., 1993). A similar study showed that TNFR1^–/–^ mice are resistant to endotoxic shock, but are not able to clear *Listeria monocytogenes* and succumb to the infection (Pfeffer et al., 1993). These studies indicate that TNFR1 plays an essential role in the host’s defense against microorganisms and their pathogenic factors. Follow-up studies showed that TNFR1 is also essential to fight *Leishmania major and Candida albicans* infections (Steinshamn et al., 1996; Nashleanas et al., 1998), indicating that TNFR1 signaling also contributes to anti-fungal and parasite defense. Mice deficient for TNFR2 also have a significant reduction in their ability to clear *C. albicans*, although in contrast to TNFR1^–/–^ mice, lethality was not increased (Steinshamn et al., 1996). Similar, in contrast to resistant wild type C57BL/6 mice, *L. major* infected TNFR2-deficient mice develop large skin lesions, which are comparable in size to those in TNFR1^–/–^ mice. However, in contrast to TNFR1^–/–^ mice, TNFR2^–/–^ mice ultimately control the infection (Fromm et al., 2015).

TNFR2 is also upregulated upon T effector cell activation (Chen et al., 2007, 2010a) and acts co-stimulatory for TCR-mediated T cell activation, as well as survival and proliferative expansion of Teff cells (Mehta et al., 2018; Ye et al., 2018). Indeed, TNFR2 expression by CD4^+^ Teffs is required to induce full-fledged experimental colitis, based on a defective proliferative expansion of TNFR2-deficient Teff cells, as well as their reduced capacity to mount a full-fledged proinflammatory Th1 cytokine response (Chen et al., 2016). Along the same line, TNFR2 was also shown to control the survival and accumulation of Teffs during the primary response against *L. monocytogenes* infection (Kim et al., 2006), indicating that TNFR2 on Teffs is important for host defense against *L. monocytogenes*. Further, sTNF-deficient transgenic mice that express a non-cleavable form of TNF were partially protected against infections with the pathogens *Mycobacterium tuberculosum* and *Listeria monocytogenes* (Torres et al., 2005; Musicki et al., 2006). Altogether, these data indicate that TNFR2 contributes to protective immune responses following infections, but, in contrast to TNFR1 is not essential for resolving the infection.

#### Non-infectious Diseases

The essential pro-inflammatory role of TNFR1 is further demonstrated by the observed decreased disease development of TNFR1^–/–^ mice in different models of non-infectious inflammatory diseases. TNFR1^–/–^ mice showed a lower incidence of disease development and an alleviated form collagen-induced arthritis (CIA) (Mori et al., 1996). However, once a joint was affected, disease severity was similar to that in wild-type mice. These data indicate that TNFR1 is the main transducer of TNF-mediated proinflammatory effects in CIA. However, the progression of arthritic disease resulting in tissue destruction and ankylosis seems to be independent of TNFR1 (Mori et al., 1996). Supporting the pro-inflammatory role of TNFR1, Deng et al., recently demonstrated that soluble versions of PLAD (sPLAD) from TNFR1 block TNF-induced responses *in vitro* and potently inhibit arthritis in animal models. In contrast, sPLAD versions from TNFR2 were less potent in inhibiting experimental arthritis (Deng et al., 2005). Because it was shown that PLADs preferentially undergo homotypic interactions, i.e., a TNFR1-sPLAD binds preferentially to a membrane expressed TNFR1, the strong therapeutic effect of TNFR1-sPLAD validates TNFR1 as a therapeutic target for arthritis and potentially other inflammatory diseases as well.

Similar to the arthritis model, TNFR1^–/–^ mice do not develop experimental autoimmune encephalomyelitis (EAE), an animal model of brain inflammation resembling MS. In contrast, TNFR2^–/–^ mice develop an exacerbated form of EAE (Eugster et al., 1999; Suvannavejh et al., 2000; Kassiotis and Kollias, 2001; Williams et al., 2014). Interestingly, it was shown that Treg-TNFR2-deficient mice develop exacerbated EAE motor disease, indicating that intrinsic TNFR2 signaling in Tregs provides protection in CNS autoimmunity (Atretkhany et al., 2018). However, another report demonstrated that TNFR2 expressed on non-hematopoietic cells is necessary for Treg function and suppression of EAE motor disease (Tsakiri et al., 2012), indicating that intrinsic and extrinsic TNFR2 activation impacts Treg functionality in EAE.

Whereas, the function of TNFR2 for nTregs is well-characterized, less is known about the impact of TNFR2 on induced Tregs (iTreg). Recently, Yang et al. (2019) demonstrated that TNFR2 deficiency impeded differentiation, proliferation, and function of iTregs. In contrast, TNFR1 deficiency resulted in reduced differentiation of inflammatory T cells, while the iTregs function was unaltered. Using a colitis model, they confirmed that TNFR2 but not TNFR1 deficiency impaired iTreg functionality (Yang et al., 2019), and proposed that TNFR2 also plays a role of iTreg function.

Next to its immunomodulatory role via Tregs, TNFR2 promotes apoptosis of insulin-specific pathogenic autoreactive CD8^+^ T cells but not normal T cells isolated from diabetes type I patients (Ban et al., 2008). Confirming, in diabetic mice administration of exogenous TNF resulted in cell death of autoreactive T cells leading to alleviation of clinical symptoms (Kodama et al., 2003). A follow-up study revealed that several defects in TNFR2-dependant activation of NFκB result in impaired anti-apoptotic effects leading to sensibilization for apoptosis (Kodama et al., 2005). Other studies showed that intrinsic TNFR2 signaling in CD4^+^ T cells impairs the differentiation of Th17 (Miller et al., 2015), outlining other potential immunomodulatory mechanisms regulated by TNFR2 signaling.

### Degenerative Diseases

Next to inflammatory diseases, where anti-TNF therapy is approved, increased levels of TNF are found in several degenerative diseases, such as heart failure (HF) or neurodegenerative diseases (Fischer and Maier, 2015; Monaco et al., 2015). Preclinical data in models of heart failure suggested that TNF neutralization in HF would be beneficial. However, clinical trials of TNF antagonists were paradoxically negative and resulted in a time- and dose-related increase in death and disease-dependent hospitalization of anti-TNF treated patients (Mann, 2002). Studies using TNFR^–/–^ mice indicate that in heart failure TNFR1 and TNFR2 induce opposing effects on tissue remodeling, hypertrophy, inflammation, and cell death. Whereas TNFR1 exacerbates these events, TNFR2 leads to amelioration of these events (Hamid et al., 2009). Other studies demonstrate that after myocardial infarction, TNFR1 activation aggravates left ventricular remodeling, whereas it is improved by TNFR2 signaling (Ramani et al., 2004; Monden et al., 2007). Altogether, these data indicate that global blocking of TNF is contraindicative for heart disease due to a protective role of TNFR2.

Similar, TNF contributes to neuropathology, i.e., it was shown that genetic overexpression of TNF in the CNS resulted in T cell infiltration, astrocytosis, and microgliosis, and chronic inflammatory demyelination (Probert et al., 1995). These studies identified TNF as an important contributor to the onset of demyelinating diseases and justified the evaluation of anti-TNF therapies in mouse models of MS. Indeed, neutralization of TNF was therapeutic in EAE mouse models of autoimmune demyelination induced by the adoptive transfer of myelin basic protein−(MBP)−sensitized T lymphocytes (Selmaj et al., 1991, 1995). However, a phase II randomized, multi-center, placebo-controlled clinical trial using the anti-TNF lenercept had to be stopped since exacerbations were significantly increased and neurologic deficits were more severe in the lenercept treatment groups compared with patients receiving placebo (Lenercept Study Group, 1999). Similar, an open-label phase I safety trial showed that two rapidly progressive MS patients showed increased MRI activity and immune activation after treatment with infliximab (van Oosten et al., 1996), and during anti-TNF therapy some juvenile RA patients developed MS-like demyelinating lesions (Sicotte and Voskuhl, 2001).

Therefore, follow-up studies using TNFR1^–/–^ and TNFR2^–/–^ mice were performed to investigate TNFR-selective responses. Interestingly, using the EAE immunization mouse modelseveral independent groups showed that TNFR1^–/–^-mice do not develop EAE motor disease, whereas TNFR2 deficiency resulted in an exacerbated form of EAE (Eugster et al., 1999; Suvannavejh et al., 2000; Kassiotis and Kollias, 2001; Williams et al., 2014), indicating opposing roles of the TNFRs in EAE. Similar results were obtained using a murine model of retinal ischemia, where TNFR1 promoted neuronal tissue destruction and TNFR2 was neuroprotective via activation of the PKB/Akt pathway (Fontaine et al., 2002).

Interestingly, compared to the vehicle group, local administration of cannabidiol after right middle cerebral artery occlusion (MCAO) resulted in reduced infarction, brain oedema and BBB permeability. Mechanistically, the group showed that cannabinoid treatment downregulated expression of TNF and TNFR1, with TNFR1 expression levels being correlated with the infarct volume (Khaksar and Bigdeli, 2017a, b). Similar studies have shown that cannabinoids inhibit inflammatory TNF activity (Rogers and Hermann, 2012; Tan and Cao, 2018), indicating that TNF/TNFR1 signaling may contribute to neurodegeneration after cerebral ischemia.

The neuroprotective role of TNFR2 was confirmed using *in vitro* studies with primary neurons. Marchetti et al. (2004) compared the impact of TNF stimulation on glutamate-induced excitotoxicity of TNFR1^–/–^ or TNFR2^–/–^ neurons. Only neurons from wild type or TNFR1^–/–^ animals were protected, while TNF activation had no protective effect on neurons from TNFR2^–/–^ mice, indicating that presence of TNFR2 was responsible for TNF-mediated neuroprotection. Mechanistically this study showed TNF-mediated neuroprotection was dependent on prolonged activation of NFκB and activation of the PI3K-PKB/Akt pathway (Marchetti et al., 2004). A follow-up study showed that TNFR2 mediates neuroprotection against glutamate−induced excitotoxicity via NFκB−dependent up−regulation of K_Ca_2.2, a member of a group of calcium-activated potassium channel known to reduce neuronal excitability (Dolga et al., 2008). Using transgenic AD mice and intracerebroventricular injection of amyloid β oligomers (AβO) into WT mice, Steeland et al. (2018) found that TNFR1 deficiency abrogated inflammation in choroid plexus and hippocampus and protected against AβO-induced morphological alterations of the choroid plexus, indicating that TNFR1 contributes to neurodegeneration.

Using the cuprizone model of toxin-induced controlled de- and remyelination, Arnett et al. (2001) demonstrated that TNFR2, but not TNFR1, is critical for oligodendrocyte regeneration. Further mechanistic studies demonstrated that astrocyte-TNFR2 promotes secretion of the chemokine Cxcl12 resulting in increased oligodendrocyte progenitor cell (OPC) proliferation and differentiation (Patel et al., 2012), supporting the remyelinating role of TNFR2. More mechanistic studies were performed using transgenic CNP-cre:TNFR2^fl/fl^ mice, where TNFR2 is selectively deleted in oligodendrocyte progenitor cells. These mice presented with exacerbated motor disease and neuropathology, including increased demyelination and reduced remyelination. This study thus shows that oligodendroglial-TNFR2 contributes to tmTNF-mediated remyelination, too (Madsen et al., 2016). Interestingly, recent work using the same animals showed that oligodendrocyte-TNFR2 not only promotes myelination, but also modulates the immune-inflammatory response in the early phase of EAE pathogenesis. In particular, specific ablation of oligodendroglial-TNFR2 resulted in increased microglia activation and blood brain barrier permeability, and accelerated infiltration of immune cells into the spinal cord prior to development of motor symptoms (Madsen et al., 2019). Further, opposing functions of microglial and macrophagic TNFR2 in the pathogenesis of EAE were reported. TNFR2-deletion in microglia resulted in increased leukocyte infiltration and demyelination into the spinal cord and early onset of motor symptoms. In contrast, TNFR2 ablation in monocytes/macrophages resulted in impaired peripheral immunity and alleviated neuropathology and EAE motor disease development (Gao et al., 2017). This work revealed an antithetic function for myeloid cells TNFR2 in EAE, with protective microglial TNFR2 signals to counteract disease development, and monocyte/macrophagic TNFR2 contributing to pathology and EAE development. These opposing effects mediated via the TNFRs indicate that inhibition of tmTNF/TNFR2 signaling was responsible for the exacerbated symptoms and may explain the failure of anti-TNF therapy in MS patients. Indeed, studies using transgenic animals that exclusively express physiologically regulated levels of tmTNF demonstrated that tmTNF is sufficient for antibacterial defense and has an important role to control chronic inflammation and autoimmunity (Alexopoulou et al., 2006).

### Chronic Neuropathic Pain

Tumor necrosis factor also plays an important role for the development of chronic neuropathic pain (CNP), a long-lasting chronic pain that is caused by damage to the somatosensory nervous system and is associated with various diseases/conditions, including neurodegenerative and inflammatory diseases, diabetes, cancer and chemotherapy (Scholz and Woolf, 2007; Murphy et al., 2017). Indeed, intra-sciatic injection of TNF in rats was shown to reproduce pain hypersensitivity similar to human neuropathic pain (Wagner and Myers, 1996; Sorkin and Doom, 2000). Studies using TNFR1/TNFR2 knock-out mice indicate that TNFR1 plays a role for death of hippocampal neurons, whereas TNFR2 played a neuroprotective role (Yang et al., 2002). However, the relative roles of TNFR1 and TNFR2 in chronic pain are still controversially discussed. TNFR1^–/–^ mice do not develop mechanical allodynia (Dellarole et al., 2014) and thermal hyperalgesia (Sommer et al., 1998), highlighting an essential role of TNFR1 for development of neuropathic pain. Interestingly, CCI did not result in pain development in male TNFR1^–/–^ mice. In contrast, female TNFR1^–/–^ mice developed CNP, however less intense than wildtype females (del Rivero et al., 2019), indicating sex-differences in TNFR1-mediated pain development.

Vogel et al. (2006) showed that thermal hyperalgesia was absent in mice deficient of TNFR1 and that both TNFR1^–/–^ and TNFR2^–/–^ mice developed an alleviated form of mechanical and cold allodynia compared to wild type mice. Another study demonstrated that TNFR1/TNFR2-double knockout mice showed reduced tactile hypersensitivity, while spontaneous pain behavior was transiently increased in a model of bone-cancer related pain. In contrast, TNFR1 or TNFR2 single knockout did not show an effect on pain sensitivity (Geis et al., 2010), indicating an interplay of TNFR1 and TNFR2 signaling for pain development in this model. In a mouse cancer model, it was shown that endogenous TNF requires TNFR2 to generate thermal hyperalgesia (Constantin et al., 2008). In particular, experimental tumor-induced thermal hyperalgesia and nociceptor sensitization were prevented by systemic administration of the anti-TNF drug etanercept. While in this model, TNFR1 gene deletion played a minor role, deletion of the TNFR2 gene reduced the painful response (Constantin et al., 2008).

In a spared nerve injury (SNI) model, immunohistochemistry analysis demonstrated that both TNFR1 and TNFR2 levels were significantly increased in the red nucleus after SNI, compared to sham-operated and normal rats (Zeng et al., 2014). A temporal analysis showed that TNFR1 expression was increased starting at 2 weeks after SNI, whereas TNFR2 expression was already elevated 1 week after injury but began to decrease by 2 weeks after injury (Zeng et al., 2014). Microinjection of anti-TNFR1 or anti-TNFR2 blocking antibodies into the red nucleus correlated with the nerve injury site increased paw withdrawal threshold in a dose-dependent manner. Combination of both anti-TNFR1 and anti-TNFR2 had the largest effect (Zeng et al., 2014). This study showed that, while TNFR1 is important throughout the development and maintenance phase of disease, TNFR2 seems to play a role for development of CNP. Similar, using a model of inflammatory pain, Zhang et al. (2011) showed that TNFR2 plays a role for mediating early-phase inflammatory pain. In particular, after intraplantar injection of complete Freund’s adjuvant (CFA), heat hyperalgesia was only alleviated early in TNFR2^–/–^ mice but reduced in both early and later phases in TNFR1^–/–^ mice (Zhang et al., 2011). In a model of experimental arthritis, chronic joint inflammation was associated with a persistent increase in TNFR1 and TNFR2 expression on dorsal root ganglion (DRG) cells. Here, after induction of arthritis, expression of TNFR1 was elevated bilaterally in neuronal cells of the DRG. In contrast, TNFR2 expression was restricted to non-neuronal cells of the macrophage-monocyte lineage that increased dependent on TNF during experimental arthritis (Inglis et al., 2005). Interestingly, the numbers of macrophages was strongly correlated to the development of mechanical hyperalgesia (Inglis et al., 2005), indicating that TNFR2-expressing macrophages may contribute to pain modulation. Summarizing, while studies demonstrate that TNFR1 plays a role for development and maintenance of neuropathic pain, the role of TNFR2 seems to be more restricted to the early phase of pain development, potentially by promoting inflammation through macrophages. Interestingly, we recently demonstrated that TNFR2^–/–^ mice have chronic non-resolving pain after CCI, a phenotype that is mirrored by depletion of Tregs (Fischer et al., 2019b), suggesting that TNFR2 may also promote analgesic responses via Tregs.

## Novel Therapeutics to Target TNFR Signaling

The activities mediated by TNFR1 and TNFR2 can be modulated in several ways and adapted to the desired therapeutic effects. Inhibition of the proinflammatory activities induced by TNFR1 can be achieved either at the level of ligand or receptor. Most of the approved therapeutics interfering with the proinflammatory activity are antibodies directed against TNF, including three IgG molecules (infliximab, adalimumab, golimumab, and several biosimilars thereof) and a PEGylated Fab fragment (certolizumab-pegol) (Kontermann et al., 2009; Monaco et al., 2015). These antibodies neutralize activation of TNFR1 and TNFR2 by inhibiting binding of TNF to its receptors, however, do not affect the activity of lymphotoxin-alpha (LTα). In contrast, a soluble TNFR2-Fc fusion protein (etanercept, and its biosimilars) is capable of inhibiting binding of TNF and LTα to its receptors (Monaco et al., 2015). Approved indications of these molecules include the treatment of chronic inflammatory diseases of the joints, digestive tract, the eye and the skin, such as rheumatoid arthritis, psoriatic arthritis, ankylosing spondylitis, Crohn’s disease, ulcerative colitis, psoriasis, hidradenitis suppurativa, uveitis, and juvenile idiopathic arthritis (Fischer et al., 2015; Monaco et al., 2015). Obviously, all these therapeutics globally affect activation of TNFR1 and TNFR2 by TNF.

**FIGURE 2:**
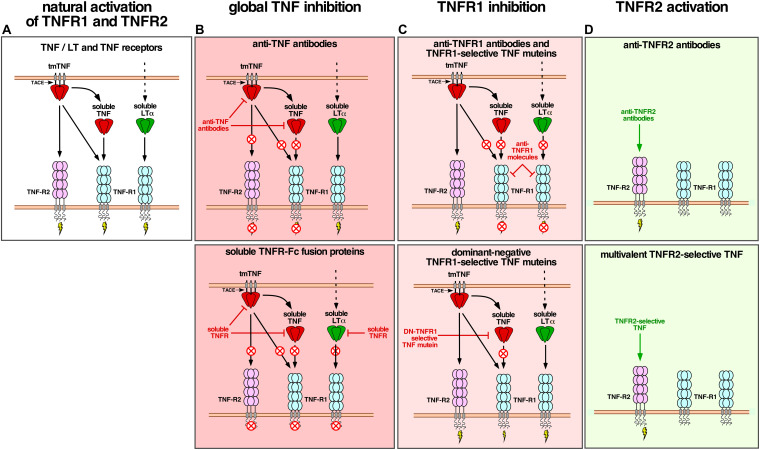
**(A)** Activation of TNFR1 and TNFR2 by membrane-bound TNF (mTNF), soluble TNF and LTα. **(B)** Global inhibition of TNFR1 and TNFR2 by anti-TNF antibodies and soluble TNFR2-Fc fusion proteins. **(C)** Selective inhibition of TNFR1 by anti-TNFR1 antibodies and dominant-negative TNFR1-selective TNF muteins. **(D)** Selective activation of TNFR2 by anti-TNFR2 antibodies and multivalent TNFR2-selective TNF muteins.

Novel therapeutics currently in development aim at a more selective inhibition of TNFR1 or are developed for a selective activation of TNFR2 (Figure 2). Selective inhibition of TNFR1 can be achieved using TNFR1 specific antibodies or modified ligands, while selective activation of TNFR2 requires (i) a specific binding to TNFR2, and (ii) the capability of activating the receptor through clustering, i.e., formation of higher order complexes (Grell et al., 1995; Fischer et al., 2017). This can be achieved using receptor-specific monoclonal antibodies or using modified ligands.

### Targeting TNFR1

**FIGURE 3:**
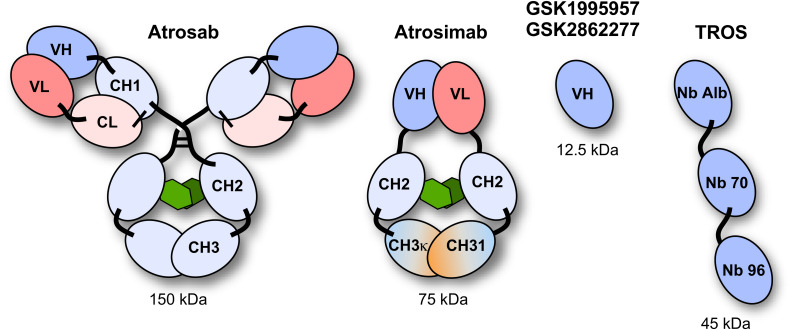
Schematic representation of the TNFR1 antagonists Atrosab (full human IgG1), Atrosimab (monovalent antibody derivate of Atrosab), GSK1995957/GS2862277 (domain antibody), and TROS (a nanobody fusion protein).

Various TNFR1-selective, neutralizing molecules have been developed in recent years, including monoclonal antibodies, antibody derivatives and TNF muteins (Figure 3). Atrosab is a humanized IgG1 derived from the mouse monoclonal antibody H398 (Kontermann et al., 2008). H398 was generated by the hybridoma technology from mice immunized with human TNFR1 and shown to compete for receptor binding with TNF and LTα (Thoma et al., 1990). Humanization was achieved by CDR grafting into human germline sequences. The humanized antibody retained the neutralizing capacity of H398 and was further developed into a human IgG1 molecule comprising an effector-deficient Fc region derived from the FcΔab sequence (Armour et al., 1999). Atrosab recognizes human and rhesus TNFR1, but not mouse TNFR1, and is capable of inhibiting TNFR1-activation by TNF and LTα with EC_50_ values in the low nanomolar range (Zettlitz et al., 2010). The epitope of Atrosab was mapped to CRD1 and CRD2 of TNFR1, with residues P23, R68, H69, located within the TNF binding site, contributing to binding (Richter et al., 2013). Atrosab could be safely administered at therapeutic doses to mice and cynomolgus monkeys and demonstrated therapeutic efficacy in various disease models (Dong et al., 2016; Williams et al., 2018). However, a first clinical phase 1 study revealed dose-limiting side effects at rather low doses, which was subsequently attributed to a marginal agonistic activity in a small concentration range observed *in vitro* due to bivalent TNFR1 binding of the IgG molecule.

This led to the development of Atrosimab, a monovalent derivative of Atrosab (Richter et al., 2019b). Atrosimab is an Fv-Fc fusion protein with approximately half the size of an antibody. The Fv fragment was generated from an alternative humanized version of H398, which was further affinity matured by CDR and random mutagenesis using phage display (Richter et al., 2019a). In order to force heterodimerization of the Fc region, a novel strategy was employed using CH3 domains engineered to comprise the CH1-CL interface of a Fab fragment. This resulted in a monovalent antibody with improved binding and neutralizing activity compared to Atrosab (Richter et al., 2019a).

Another monovalent anti-TNFR1 binder was generated using a single antibody heavy chain domain (VH; domain antibody − dAb), which acts a competitive antagonist and lacks agonistic activity (Holland et al., 2013). This molecule was developed by GSK (GSK1995057) and had entered preclinical and clinical testing, including i.v. and pulmonary delivery (Proudfoot et al., 2018). Surprisingly, a novel type of autoantibody (HAVH) reacting with the human VH framework used in GSK1995957 was identified in approximately 50% of healthy human serum samples. *In vitro* studies showed that these pre-existing anti-drug antibodies led to TNFR1 activation and infusion reactions consistent with cytokine release, limiting its therapeutic use (Holland et al., 2013; Cordy et al., 2015). Information derived from the HAVH binding epitope on the VH was used to generate a derivative (GSK2862277) with reduced binding of HAVH autoantibodies reducing the frequency of donors with pre-existing autoantibodies to 7%. However, in a phase 1 trial adverse effects due to presence of high levels of novel pre-existing antibodies were observed in one subject (Cordy et al., 2015). Another obstacle for use in patients comes from the rather short serum half-life of these domain antibodies with a size of approximately 10−13 kDa. This can be circumvented by implementing half-life extension strategies (Kontermann, 2011). In one approach, an anti-mouse TNFR1 domain antibody (DOM1m-21-23) was fused to an albumin-binding domain antibody, resulting in a bispecific fusion protein (DMS5540) which showed dose-depended extension of half-life in mice (from 3.3 h at 0.1 mg/kg to 23.2 h at a dose of 10 m/kg), indicative of target-mediated clearance. Furthermore, protective activity in a prophylactic mouse challenge study with bolus injected TNF was observed starting with doses of 0.3 mg/kg (Goodall et al., 2015).

Similarly, two anti-TNFR1 Nanobodies (Nb) isolated from an alpaca immunized with recombinant human soluble TNFR1 were genetically linked to an albumin-binding Nb to generate a bispecific half-life extended molecule named “TNF Receptor-One Silencer” (TROS) (Steeland et al., 2015). TROS competes with TNF for binding to TNFR1, inhibits its activity with IC_50_ values in the nanomolar range and showed therapeutic activity in *ex vivo* and *in vivo* models of inflammation, e.g., in a EAE model in human TNFR1 transgenic in a mouse TNFR1-k/o-background (Steeland et al., 2017). In these mice, TROS exhibited a serum half-life of >24 h after i.p., injections requiring administration every 2nd day.

Others have developed small molecular inhibitors of TNFR1, including antisense oligonucleotides (ASO) and small-molecule inhibitors identified by screening compounds of the NIH Clinical Collection (Lo et al., 2017, 2019). The ASO approach was used to induce downregulation of TNFR1, allowing tumor therapy with high dose TNF, i.e., protecting animals from systemic TNF-induced toxicity (van Hauwermeiren et al., 2015). The small-molecule inhibitors either disrupted the interaction of the TNFR1 pre-ligand assembly domain (PLAD) or acted allosterically on TNFR1 (Lo et al., 2017). In a recent study, the cheminformatics pipeline was used to identify compounds in the Zinc database that inhibit TNFR1 using a pharmacophore-based screening, molecular docking and *in silico* ADMET (absorption, distribution, metabolism, excretion and toxicity) prediction (Saddala and Huang, 2019).

The use of TNF muteins represents another approach for selective interference with TNFR1 activity. R1antTNF is a modified TNF with specificity for TNFR1 isolated from a TNF phage display library (Shibata et al., 2008a). This TNF mutein, with an affinity for TNFR1 similar to that of the wild-type TNF, carries the mutations A84S, V85T, S86T, Y87H, Q88N, and T89Q, and inhibits TNFR1-mediated activity without affecting TNFR2. It was reasoned from x-ray crystallographic studies that one of the mutations, Y87H, which changes the binding mode from a hydrophobic to an electrostatic interaction, causing an unstable, rapid TNFR1 binding pattern, is responsible for the antagonistic activity (Shibata et al., 2008b), which was also confirmed for another TNF mutein, R1antTNF-T8, bearing in addition a T89R mutation (Mukai et al., 2009). Therapeutic activity of R1antTNF was demonstrated in various animal models. Another study revealed that R1antiTNF binds TNFR1 with fast association and dissociation rates, resulting in a shortened nuclear duration of NFκB and a gene expression profile biased toward early response genes (Zhang et al., 2017). Interestingly, at higher concentrations R1antTNF selectively activates the apoptosis pathway and not the NFκB pathway. Half-life of this short-lived TNF mutein was improved through PEGylation (PEG-R1antTNF), which improved furthermore the therapeutic activity, e.g., in an EAE model of MS (Nomura et al., 2011). Furthermore, the molecular stability and bioactivity was improved by converting the homotrimeric R1antTNF into a single-chain derivative (scR1antTNF) by introducing short peptide linkers of 5 or 7 residues between the three protomers (Inoue et al., 2017).

One of the most studied TNF muteins is XPro1595 and a PEGylated derivative thereof. XPRo1595 is a dominant-negative mutant of TNF developed by Xencor applying an *in silico* method to predict and design homotrimeric TNF variants exhibiting decreased receptor binding and being capable of sequestering native TNF homotrimers into inactive native:variant heterotrimers leading to inhibition of TNF-mediated signaling (Steed et al., 2003). XPro1595 carries two mutations, A145R and Y87H, located at the TNF-TNFR interface, and is unable to bind TNFR1 or TNFR2 and to activate downstream signals as homotrimer. XPro1595 efficiently blocks the activity of TNF by exchanging individual subunits and forming heterotrimers. Thus, exchange of one subunit already leads to an inactive TNF molecule, which can bind only one TNF receptor chain, insufficient for receptor activation. At a ratio of 10:1 XPro1595 to wt TNF already 99% of the TNF molecules are inactivated. XPro1595 was further modified into a PEGylated derivative (XENP1595) for increased half-life and reduced immunogenicity. This involved the introduction of three mutations, C69V, C101A, and R31C, allowing a site-directed PEGylation at C31 (Zalevsky et al., 2007; Olleros et al., 2009). Of note, membrane TNF is not affected by XPro1595 and its derivatives.

### Targeting TNFR2

Besides selective binding to TNFR2, activation of TNFR2 requires efficient receptor clustering, which is mediated by membrane TNF or secondary receptor cross-linking, e.g., with anti-TNFR2 antibodies (Wajant et al., 2001). Various TNF muteins selectively binding to TNFR2 have been generated by site-directed mutagenesis or using phage display (Loetscher et al., 1993; Abe et al., 2011; Ando et al., 2016). One of the most commonly used variant is a double mutation in human TNF, D143N and A145R, which lacks complete binding to human or mouse TNFR1 (Loetscher et al., 1993). The soluble, homotrimeric TNF molecule comprising the receptor-binding TNF-homology domain (aa 80 - 233) was further converted into a single-chain derivative (scTNF) by connecting the three subunits (protomers) with 2 flexible linkers, e.g., composed of glycines and serines (Krippner-Heidenreich et al., 2008). This increased the stability under physiological conditions *in vitro* and *in vivo*, while maintaining receptor selectivity. Furthermore, the scTNF moiety allows to generate fusion proteins to increase valency for TNFR2. One of the first approached was fusion of the trimerization of domain of tenascin C (TNC) to the N-terminus of TNFR2-selective scTNF, resulting in a nonavalent molecule capable of clustering TNFR2 (Fischer et al., 2011b). This approach was also applied to generate a mouse TNFR2-selective mouse TNC-scTNF. Here, two mutations, D221N and A223R, were introduced into mouse TNF (Fischer et al., 2014; Chopra et al., 2016). In an alternative approach, the homodimerization heavy chain domain 2 of human IgE (EHD2) was used to generate a hexavalent fusion protein (EHD2-scTNF_R__2_), which was also capable of specifically binding to TNFR2 and inducing efficient receptor activation (Dong et al., 2016). Furthermore, the use of tetramerization domains, e.g., derived from p53 and GCN4, was applied to obtain dodecavalent fusion proteins with further improved crosslinking activity (Fischer et al., 2017). The use of Fc-regions or whole antibodies represents another option to generate hexavalent molecules and, in addition, allows to obtain targeted derivatives, e.g., as has been shown for scTRAIL fusion proteins (Hutt et al., 2018; Siegemund et al., 2018).

Selective TNFR2 activation was also described for a homotrimeric TNF variant (TNF07) carrying 2 mutations, S95C and G148C, which result in disulfide-linked TNF molecules with increased stability and, surprisingly, the capability to activation TNFR2 without further crosslinking, as shown in CD4^+^ T-regulatory expansion assays, although the molecular composition and absence of TNF07 multimers was not analyzed (Ban et al., 2015).

TNFR2 agonism can also be induced by TNFR2-selective antibodies. Screening available anti-TNFR2 monoclonal antibodies, one agonistic antibody was identified leading, e.g., in activation and expansion of T_reg_ cells, capable of correcting type 1 diabetes-associated Treg activation defects (Okubo et al., 2013, 2016). Mechanistically, it has been proposed that antagonistic anti-TNFR2 antibodies block ligand binding and lock membrane receptors in a resting (non-signaling), antiparallel dimer arrangement, while agonistic, cross-linking antibodies stabilize parallel TNF-TNFR2 complexes, i.e., provide a structural stabilization of the active signaling network (Vanamee and Faustman, 2018). Ligand-independent activation of TNFR2 by antibodies can, furthermore, be induced by Fc-mediated binding to FcγR on neighboring cells resulting in multivalent membrane display, thus mimicking membrane TNF. A potent, Fc-dependent T-cell co-stimulation and robust antitumor effects of these type of antibodies were described (Tam et al., 2019). Alternatively, combining an anti-TNFR2 antibody with an anchoring domain mediating binding to a membrane protein has also been described to allow a FcγR-independent TNFR2 activation. This was exemplarily shown fusing co-stimulatory members of the TNFSF, such as scGITRL, sc4-1BBL and IL-2, to the C-terminus of an anti-TNFR2 monoclonal antibody (Medler et al., 2019). Similarly, bispecific antibodies could be used to retarget the TNFR2 binding site and to induce a multivalent presentation, as shown for a tetravalent bispecific anti-TRAILR2 antibody targeting fibroblast activation protein (FAP) on tumor stroma fibroblasts (Brünker et al., 2016).

## Selective Neutralization of sTNF by Dominant-Negative TNF Muteins

Since tmTNF is sufficient to promote important immune functions like self-tolerance and resistance to infection (Alexopoulou et al., 2006), selective neutralization of sTNF may be a superior therapeutic strategy to treat chronic inflammatory and autoimmune diseases compared to non-selective blocking of TNF. The dominant-negative TNF mutein XPro1595 has shown therapeutic activity in disease models of inflammatory and degenerative diseases. It was first shown in 2007 that sTNF neutralization attenuates experimental arthritis in two rodent arthritis models without suppressing innate immunity to *Listeria* infection (Zalevsky et al., 2007), indicating that inflammation in mouse arthritis models is primarily driven by sTNF, and suggest that sTNF inhibitors might have a superior safety profile compared to conventional non-selective anti-TNF therapeutics. A follow-up study using XPro1595 showed that selective inhibition of sTNF protected mice from Bacillus Calmette-Guérin (BCG)/LPS and D-GALN/LPS-induced liver damage, indicating that sTNF, but not tmTNF, is critical for LPS-induced hepatitis (Olleros et al., 2010).

The main focus of pre-clinical studies using DN-TNFs is on treatment of neurodegenerative diseases where elevated TNF levels are found at the site of injuries, such as MS, Parkinson’s disease (PD) and spinal cord injury (SCI). XPro1595 was evaluated in two parallel studies by the groups of Lesley Probert and John Bethea in the EAE mouse model of MS. Both studies showed that blocking the action of sTNF by XPro1595, but not of sTNF/tmTNF by the non-selective anti-TNF therapeutic etanercept, protected mice against the clinical symptoms of EAE. Taoufik et al. (2011) treated at time of disease onset and demonstrated that the therapeutic effect in this study was associated with reduced CNS immunoreactivity and increased expression of neuroprotective mediators but independent of changes in antigen-specific immune responses and focal inflammatory spinal cord lesions, but was (Taoufik et al., 2011). Brambilla et al. (2011) treated the EAE mice at peak of disease, when marked demyelination was already in progress, and showed that XPro1595 administration resulted in reduced axon damage, preservation of axons and improved myelin compaction and significant remyelination. Mechanistic studies showed that therapeutic inhibition of soluble brain TNF promotes remyelination due to improved phagocytosis of myelin debris by microglia and prevented disease-associated decline in motor performance in cuprizone-fed mice (Karamita et al., 2017). These results demonstrate that sTNF promotes CNS inflammation in EAE and indicate that blocking of neuroprotective tmTNF might have been the cause of the failed lenercept trial.

The laboratory of Malú Tansey has demonstrated that blocking sTNF signaling attenuates loss of dopaminergic neurons in models of Parkinson’s disease. Local administration of the dominant-negative TNF inhibitor XENP345, an earlier version of XPro1595 that works via the same mechanism of action, reduced the retrograde nigral degeneration induced by a striatal injection of the oxidative neurotoxin 6-hydroxydopamine (6-OHDA) by 50%. Similar neuroprotective effects were observed after chronic co-infusion of XENP345 with bacterial lipopolysaccharide (LPS) into the substantia nigra (McCoy et al., 2006). Another study from the same laboratory showed that intranigral lentiviral delivery of dominant-negative TNF administered concomitant with 6-OHDA attenuated neurotoxin-induced DA neuron loss and associated behavioral deficits in hemiparkinsonian rats (McCoy et al., 2008). Similar, delayed injection of DN-TNF encoding lentivirus 2 weeks after receiving a 6-OHDA lesion attenuated microglia activation and halted progressive loss of nigral dopaminergic neurons (Harms et al., 2011). Interestingly, peripheral administration of XPro1595 resulted in significant CSF levels of the TNF mutein and attenuated glial activation and nigral cell loss and in 6-OHDA hemiparkinsonian rats (Barnum et al., 2014). Collectively, these data clearly demonstrate a role for sTNF in PD pathology, and indicate that selective inhibition of sTNF may be therapeutic in early stages of PD.

Other work from the Tansey laboratory indicates the therapeutic potential of XPro1595 for Alzheimer’s disease (AD). Using 5xFAD mice, which express human *a*myloid precursor protein (*APP*) and presenilin-1 (PSEN1) transgenes and recapitulate many AD-related phenotypes, they showed that peripheral injection of XPro1595 alleviated the age-dependent increase in activated immune cells in the brain of transgenic mice, decreased beta-amyloid plaque load, and rescued impaired long-term potentiation (LTP). This indicates that sTNF neutralization may impact brain immune cell infiltration and prevent or delay neuronal dysfunction in AD (MacPherson et al., 2017). Similar, chronic infusion of XENP345 or single injection of a lentivirus encoding DN-TNF abrogated AD-like pathology in LPS-treated 3xTgAD mice (McAlpine et al., 2009). Further data indicate that XPro1595 administration lowers the risk for late-onset Alzheimer’s disease associated with obesity, metabolic syndrome, and type 2 diabetes (Sousa Rodrigues et al., 2019).

Interestingly, genetic ablation of sTNF did not reduce lesion size and improve functional recovery after moderate SCI in mice (Ellman et al., 2016). In contrast, epidural administration of XPro1595 to the contused spinal cord decreased anxiety-related behavior, and reduced neuronal damage at the site of injury resulting in improved locomotor function, whereas central administration of the non-selective anti-TNF drug etanercept had no therapeutic effects (Novrup et al., 2014). Further studies in rats demonstrated that intrathecally administered XPro1595 directly post-high-level SCI improved the intensification of colorectal distension-induced and naturally occurring autonomic dysreflexia, a life-threatening syndrome experienced by SCI patients. This effect was mediated via decreased sprouting of nociceptive primary afferents and activation of the spinal sympathetic reflex circuit (Mironets et al., 2018). A follow-up study from the same laboratory further demonstrated that delayed (3 days after injury) local administration of XPro1595 still improved autonomic dysreflexia for months postinjury. Further, XPro1595 administration also prevented sympathetic hyperreflexia-associated splenic atrophy and loss of leukocytes to dramatically improve the ability of chronic SCI rats to fight off pneumonia, a common cause of hospitalization after injury (Mironets et al., 2020). Interestingly, subcutaneous administration of XPro1595 caused an exacerbation of SCI-associated depressive phenotype in rats, whereas intracerebroventricular administration of the drug did not impact the development of depression after injury (Farrell and Houle, 2019). This suggests a complex contribution of TNF-based neuroinflammation in SCI−induced depression.

Clausen et al. studied systemic administration of Xpro1595 and etanercept on infarct volume, functional recovery and inflammation after focal cerebral ischemia in mice. They found that systemically administered XPro1595 and etanercept significantly improved functional outcomes, such as brain inflammation and liver acute phase response (APR), but did not affect infarct volumes (Clausen et al., 2014). In a follow-up study, mice were treated topically or intracerebroventricularly with saline, XPro1595, or etanercept immediately after permanent MCAO. Topical, but not intracerebroventricular XPro1595 treatment reduced infarct volume after pMCAO, whereas etanercept administration had no effect (Yli-Karjanmaa et al., 2019). Altogether, these data indicate that inhibition of sTNF signaling holds promise as a novel treatment for ischemic stroke.

Genetic data indicate that TNFR1 plays an essential role for pain development in males (Dellarole et al., 2014). Accordingly, it was shown that intraperitoneal administration of XPro1595 prevented complete Freund’s adjuvant (CFA)-induced mechanical hypersensitivity in male mice in a model of local CFA-induced model of orofacial pain (Lis et al., 2017). Similar, after CCI, systemic application of XPro1595 alleviated mechanical allodynia in males. However, no therapeutic response was observed in females. Mechanistically this study showed that presence of estrogen inhibited the therapeutic response of XPro1595 in females, i.e., XPro1595 was therapeutic in ovariectomized mice, whereas the therapeutic effect was lost after estrogen replacement therapy in ovariectomized mice (del Rivero et al., 2019). This study indicates sex-difference in the response to DN-TNFs. Since most disease models are limited to analysis of one sex, further investigations are needed to evaluate sex differences in other disease models, such as EAE or PD/AD models.

Shibata et al. (2008a) studies the therapeutic effect of R1antTNF in chemically induced acute hepatitis models. In a carbon tetrachloride (CCl_4_)-induced model, R1antTNF administration significantly reduced serum levels of ALT (alanine aminotransferase), a marker for liver damage. In a concanavalin A (ConA)-induced T-cell-dependent model, R1antTNF administration reduced serum levels of the inflammatory cytokines IL-2 and IL-6 (Shibata et al., 2008a). Importantly, the efficacy of R1antTNF treatment was superior to antagonistic anti-TNF antibodies, indicating that blocking of TNFR1 might be superior to non-specific neutralization of sTNF/tmTNF. The therapeutic effect of pegylated R1-antTNF was then evaluated in animal models of chronic inflammation. In a murine collagen-induced arthritis model XPro1595 showed a comparable therapeutic effect to etanercept in a prophylactic treatment setting. However, in therapeutic protocols, PEG-R1antTNF showed a greater therapeutic effect than etanercept. Moreover, PEG-R1antTNF did not affect the clearance of injected adenovirus. In contrast, virus load strongly accumulated during etanercept treatment (Shibata et al., 2009). Further, PEG-R1antTNF treatment at time of disease induction significantly improved the clinical score and suppressed peripheral and central Th1 and Th17-type response as well as cerebral demyelination in EAE mice (Nomura et al., 2011). Similar, PEG-R1antTNF treatment attenuated arterial inflammation and intimal hyperplasia in IL-1 receptor antagonist-deficient mice (Kitagaki et al., 2012). Altogether, these data indicate that inhibition of sTNF/TNFR1 seems to be superior to unspecific sTNF/tmTNF neutralization by conventional anti-TNF drugs (Table 1).

**TABLE 1:** Preclinical Use of sTNF neutralizing therapeutics.

Molecule	Disease model	References
**Dominant-negative TNF muteins (DN-TNF)**		
XENP345/XPro1595	Experimental arthritis	Zalevsky et al., 2007
XPro1595	(BCG)/LPS and D-GALN/LPS-induced liver damage	Olleros et al., 2009, 2010
XPro1595	Experimental autoimmune encephalomyelitis (EAE)	Brambilla et al., 2011; Taoufik et al., 2011; Karamita et al., 2017
XENP345, lentiviral DN-TNF delivery, XPro1595	6-OHDA- and LPS-induced models of Parkinson disease	McCoy et al., 2006, 2008; Harms et al., 2011; Barnum et al., 2014
XPro1595	5xFAD transgenic mice as a model of Alzheimer’s disease	MacPherson et al., 2017
XENP345, lentiviral DN-TNF delivery	LPS-treated 3xTgAD transgenic mice as a model of Alzheimer’s disease	McAlpine et al., 2009
XPro1595	high-fat high-carbohydrate diet induced model of insulin impairment	Sousa Rodrigues et al., 2019
XPro1595	Spinal cord injury: motor impairment	Novrup et al., 2014
XPro1595	Spinal cord injury: autonomic dysreflexia and antibacterial immunity	Mironets et al., 2018, 2020
XPro1595	Focal cerebral ischemia: neuroinflammation and liver acute phase response	Clausen et al., 2014
XPro1595	Permanent Middle Cerebral Artery Occlusion (pMCAO): infarct volume	Yli-Karjanmaa et al., 2019
XPro1595	CFA-induced orofacial pain	Lis et al., 2017
XPro1595	Chronic constriction injury (CCI)	del Rivero et al., 2019
R1antTNF	CCl_4_- and ConA-induced hepatitis	Shibata et al., 2008a
PEG-R1antTNF	Collagen-induced arthritis	Shibata et al., 2009
PEG-R1antTNF	Experimental autoimmune encephalomyelitis (EAE)	Nomura et al., 2011
PEG-R1antTNF	femoral artery injury in IL1R-deficient mice: arterial inflammation and intimal hyperplasia	Kitagaki et al., 2012

## Blocking of TNFR1 by TNFR1-Selective Antagonists

To neutralize pro-inflammatory TNFR1 signaling, we have developed the human TNFR1 specific antagonist Atrosab. Similar to sTNF neutralization, Atrosab ameliorated EAE motor disease. To study long-term efficacy of TNFR1 antagonist treatment the parental mouse anti-human TNFR1 antibody H398 was administered. Interestingly, our data indicate that TNFR1 blocking restricts CNS-infiltration of peripheral immune cell through down-regulation of TNF-induced adhesion molecules and not by impacting peripheral immunity (Williams et al., 2018). Further, in a CIA rhesus monkey model, Atrosab administration resulted in reduced acute-phase C-reactive protein (CRP) and IL-6 levels in serum, prevented body weight loss, delayed the onset of arthritic symptoms and improved the clinical arthritis score (Guenzi et al., 2013). Moreover, therapeutic efficacy of Atrosab was superior to the clinically used anti-TNF drugs etanercept and infliximab (Guenzi et al., 2013). Importantly, using a mouse model of NMDA-induced acute neurodegeneration, we demonstrated that co-administration of Atrosab together with glutamate into the magnocellular nucleus basalis resulted in protection of cholinergic neurons from glutamate-induced excitotoxic cell death and reverted the neurodegeneration-associated memory impairment tested by a passive avoidance paradigm (Dong et al., 2016). Interestingly, administration of Atrosab together with a TNFR1 antagonist abrogated the therapeutic effect of Atrosab, indicating that the therapeutic activity of Atrosab depends on functional TNFR2 signaling, which appears essential for neuroprotection (Dong et al., 2016).

Recently, we further demonstrated that Atrosab might be a promising novel therapeutic for non-alcoholic fatty liver disease (NAFLD), a wide-spread disease with increasing prevalence that is associated with the development of liver fibrosis/cirrhosis, a major risk factor of liver-related and all-cause mortality in this disease (Chalasani et al., 2018). Activation of pro-inflammatory cytokines, such as TNF, in adipose and liver tissues has been implicated to play an important role in the pathogenesis and disease progression of NAFLD (Hotamisligil et al., 1993; Crespo et al., 2001). Indeed, higher serum levels of TNF correlate with insulin resistance patients and were observed in samples from non-alcoholic steatohepatitis (NASH) patients compared to samples from patients with simple steatosis (Hui et al., 2004; Wellen and Hotamisligil, 2005). Moreover, in liver tissues of NASH patients enhanced TNF/TNFR1 expression was found in correlation with disease activity and fibrosis stages (Crespo et al., 2001). Vice versa, in various diet-induced or genetic NAFLD models, TNF- or TNFR-deficient mice showed improved insulin sensitivity and less pronounced liver steatosis and fibrosis (Uysal et al., 1997, 1998; Tomita et al., 2006). Our data show that blocking of TNFR1 by Atrosab results in alleviation of liver steatosis and insulin resistance as well as liver injury and fibrosis (Wandrer et al., 2020). Selective TNFR1 inhibition might therefore represent a promising treatment strategy in NAFLD.

The nanobody-based selective inhibitor of TNFR1 TROS reduced secretion of IL-6, IL-8 and TNF in *ex vivo* cultured inflamed colon biopsies from patients suffering from active Crohn’s disease. Similar, in liver chimeric humanized mice, TROS antagonized inflammation in a model of acute TNF-induced liver inflammation (Steeland et al., 2015). The neuroprotective effect of TROS was affirmed using transgenic AD mice and icv injection of AβO into WT mice. Here, Steeland et al. (2018) showed that therapeutic blockage of TNFR1 by TROS prevented the cognitive decline in APP/PS1^tg/wt^ mice and upon icv AβO injection, outlining the therapeutic potential of TNFR1 antagonists for AD. Similar to Atrosab, TROS was therapeutic in a model of MS. It was shown that prophylactic TROS treatment significantly delayed disease onset and ameliorated EAE symptoms in mice. Treatment initiated early after disease onset prevented further disease development. Altogether, TROS administration reduced neuroinflammation and preserved myelin and neurons (Steeland et al., 2017). The therapeutic responses of TROS and Atrosab in EAE indicate that TNFR1 blocking might be therapeutic in MS. Indeed, through genome-wide association studies, a single nucleotide polymorphism (SNP) in the TNFRSF1A gene encoding TNFR1 was discovered to be associated with MS, but not with other autoimmune conditions such as rheumatoid arthritis, psoriasis and Crohn’s disease. Functional studies showed that this MS risk allele directs expression of a novel, soluble form of TNFR1 that can neutralize TNF, similar to anti-TNF therapeutics (Gregory et al., 2012). Together with the overwhelming data describing TNFR2 as an essential mediator of neuroprotection this indicated that maintenance of functional TNFR2 signaling is important during MS therapy. Therefore, selective blocking of TNFR1 might be superior to anti-TNF therapeutics like lenercept, which failed in clinical trials of MS (Table 2).

**TABLE 2:** Preclinical Use of TNFR1 blocking therapeutics.

Molecule	Disease model	References
**TNFR1 blocking reagents**		
Atrosab	Experimental autoimmune encephalomyelitis (EAE)	Williams et al., 2018
Atrosab	Collagen-induced arthritis	Guenzi et al., 2013
Atrosab	NMDA-induced neurodegeneration model of Alzheimer’s disease	Dong et al., 2016
Atrosab	non-alcoholic steatohepatitis (NASH)	Wandrer et al., 2020
TROS	Acute TNF-induced liver inflammation	Steeland et al., 2015
TROS	AβO injection into APP/PS1^tg/wt^ mouse model of Alzheimer’s disease	Steeland et al., 2018
TROS	Experimental autoimmune encephalomyelitis (EAE)	Steeland et al., 2017

## Selective Activation of TNFR2 Using Agonistic TNF Muteins and Antibodies

TNFR2 agonist may work via a dual mode of action, modulation of immunity and direct neuroprotection. Therefore, TNFR2 agonists were evaluated in models of inflammation and neurodegeneration (Table 3). Indeed, several articles using different TNFR2 agonists demonstrated that TNFR2 activation results in expansion of Tregs *ex vivo* and *in vivo* (Okubo et al., 2013; Chopra et al., 2016; Fischer et al., 2017, 2018, 2019a,b). Using the mouse TNFR2 agonist STAR2, Chopra et al. (2016) showed that exogenous TNFR2 activation protected from acute graft-versus-host disease (GvHD) after allogeneic hematopoietic stem cell transplantation (allo-HCT) via host Treg cell expansion. In this model, Tregs were first expanded via STAR2 administration in recipient mice before allo-HCT, which led to a significantly prolonged survival and reduced GvHD severity in a TNFR2- and Treg-dependent manner. Importantly, the beneficial effects of transplanted T cells to attack leukemic cells and infectious pathogens remained unaffected (Chopra et al., 2016). Another study using a human TNFR2 selective STAR2 variant demonstrated that TNFR2 impeded differentiation of bone marrow-derived immature myeloid cells in culture and dampened their suppressor function *in vitro*. *In vivo* administration of STAR2 resulted in mild myelopoiesis in naïve mice but did not affect immune cell composition. In mice with chronic inflammation, STAR2 treatment expanded CD4^+^ Tregs and improved their suppressive function (Schmid et al., 2017).

**TABLE 3:** Preclinical Use of TNFR2 agonists and antagonists.

Molecule	Disease model	References
**TNFR2 agonists**		
STAR2	Graft versus host disese (GvHD)	Chopra et al., 2016
STAR2, EHD2-sc-mTNF_R2_	Collagen-induced arthritis	Fischer et al., 2018; Lamontain et al., 2019
EHD2-scTNF_R2_	NMDA-induced neurodegeneration model of Alzheimer’s disease	Dong et al., 2016
EHD2-sc-mTNF_R2_	Spinal cord injury (SCI)	Gerald et al., 2019
EHD2-sc-mTNF_R2_	Chronic constriction injury (CCI) model of neuropathic pain	Fischer et al., 2019b
EHD2-sc-mTNF_R2_	Experimental autoimmune encephalomyelitis (EAE)	Fischer et al., 2019a
Y9 (agonistic anti-TNFR2 antibody)	Syngeneic mouse tumor models	Tam et al., 2019
**TNFR2 antagonists**		
TNFR2 antagonistic antibodies	Ovarian cancer (patient material)	Torrey et al., 2017
TNFR2 antagonistic antibodies	Sézary syndrome (patient material)	Torrey et al., 2019

Using the mouse TNFR2 agonist EHD2-sc-mTNF_R__2_, we demonstrated that selective activation of TNFR2 induces anti-inflammatory responses and alleviates experimental arthritis. Interestingly, we observed that TNFR2 agonism expands both CD4^+^ and CD8^+^ FoxP3^+^ Tregs both *ex vivo* and in CIA mice (Fischer et al., 2018). This might be important for the therapeutic effect of TNFR2 agonists, since CD8^+^ suppressor cells were shown to be more suppressive in arthritic mice than their CD4^+^ counterparts (Notley et al., 2010). In the applied 10-day observation protocol, we only observed a therapeutic response by EHD2-sc-mTNF_R__2_ in a prophylactic setting, whereas treatment after onset of arthritis did not impact arthritic disease within the observation period. However, another study using STAR2 in CIA mice showed that TNFR2 agonist treatment ameliorates established collagen-induced arthritis in mice (Lamontain et al., 2019). Of note, in this protocol, TNFR2 agonist treated mice showed amelioration of arthritic disease only after more than 10 days observation period. Together, these two independent studies suggest a therapeutic potential of TNFR2 agonists for arthritis and other chronic inflammatory diseases.

Using EHD2-scTNF_R__2_ we confirmed the neuroprotective role of TNFR2 and demonstrated that selective activation of TNFR2 rescued dopaminergic neurons (Fischer et al., 2011b) and oligodendrocytes (Maier et al., 2013) from oxidative stress induced cell death and promoted myelination via astrocyte-dependent secretion of neurotrophic factors (Fischer et al., 2014). In this line, we showed that coadministration of glutamate and EHD2-scTNF_R__2_ into the magnocellular nucleus basalis of mice protected cholinergic neurons and their cortical projections from excitotoxic cell death induced by glutamate and reverted the injury-associated memory impairment testes by a passive avoidance paradigm (Dong et al., 2016). Similar, using a mouse model of contusive injury, Gerald et al. showed that EHD2-sc-mTNF_R__2_-mediated activation of TNFR2 in the spinal cord improved locomotion and cortical neural activity (Gerald et al., 2019). Due to the important role of TNFR2 for neuroprotection, we went on to study the neuroprotective role of TNFR2 in models of CNP. Here, we showed that pharmacological activation of TNFR2 using EHD2-sc-mTNF_R__2_ in mice promoted long-lasting pain recovery after CCI. TNFR2 agonist treatment alleviated peripheral and central inflammation and reduced neuronal injury. Importantly, depletion of Tregs abolished the therapeutic effect of TNFR2 agonist treatment (Fischer et al., 2019b), indicating that Treg-TNFR2 mediated responses are essential for the analgesic effect of EHD2-sc-mTNF_R__2_. Similar, we demonstrated that in EAE mice systemic administration of EHD2-sc-mTNF_R__2_ alleviated inflammation resulting in reduced demyelination and neurodegeneration. The behavioral data showed that TNFR2 agonist treatment alleviated motor disease and promoted long-term recovery from CNP. Mechanistically, this study indicated that TNFR2 agonist treatment in EAE mice follows a dual mode of action and promotes suppression of CNS autoimmunity as well as remyelination (Fischer et al., 2019a).

The group of Denise Faustman used an agonistic TNFR2-selective antibody to demonstrate that a subpopulation of insulin-specific CD8^+^, but not CD4^+^, T cells in blood samples from patients with type 1 diabetes was vulnerable to TNFR2 induced death. However, other activated and memory T cell populations were resistant to TNFR2-triggered cell death (Ban et al., 2008). This indicates that autoreactive T cells in type 1 diabetes patients can be selectively destroyed by TNFR2 agonism. TNFR2 agonist may offer highly targeted therapies, with a potentially reduced risk of systemic toxicity. Using their agonistic αTNFR2 antibody, Okubo et al. further established a protocol for homogenous expansion of Tregs from human donors (Okubo et al., 2013). Therefore, TNFR2 agonists might work via two different mode of action in diabetes, killing of autoreactive T cells and expansion of immunomodulatory Tregs.

## Selective Modulation of TNFR2 Signaling for Cancer Therapy

Next to its potential use as a therapeutic target in inflammatory and degenerative diseases, TNFR2 was recently identified as a novel drug target for the treatment of cancer (Table 3). Next to its function on immunosuppressive Tregs and myeloid-derived suppressor cells, which may inhibit immune responses to combat tumor development, TNFR2 is expressed on certain tumor cells and directly promotes their proliferation (Vanamee and Faustman, 2017; Sheng et al., 2018). Indeed, TNFR2 plays important roles in multiple aspects of tumor progression, including tumor cell proliferation, bypassing of immune surveillance, promotion of angiogenesis, the formation of a pre-metastasis milieu (reviewed in Sheng et al., 2018). Therefore, therapeutic strategies targeting TNFR2-mediated tumor growth include depletion of TNFR2-expressing Tregs (van der Most et al., 2009) and antagonistic antibodies targeting TNFR2 over-expressed on tumor cells. Several antagonistic antibodies were shown to directly kill human ovarian tumor cells and Tregs by blocking ligation of TNF to TNFR2. Importantly, these antagonistic TNFR2 antibodies depleted Tregs isolated from ovarian cancer ascites more potently than Tregs from healthy donor samples, implying increased tumor specificity (Torrey et al., 2017). A follow-up study indicated that targeted killing of TNFR2-expressing tumor cells and Tregs using TNFR2 antagonistic antibodies is therapeutic in advanced Sézary syndrome, a rare form of cutaneous T-cell lymphoma that is often refractory to treatment (Torrey et al., 2019). Interestingly, next to TNFR2 antagonists, agonistic monoclonal anti-TNFR2 antibodies yielded robust antitumor activity and durable protective antitumor immunity in multiple mouse cancer cell line models. These antibodies mediated potent Fc-dependent T cell co-stimulation but did not impact numbers or function of Tregs (Tam et al., 2019). These and other studies indicate the complex role of TNFR2 for tumor growth and therapy and suggest that selection of a therapeutic approach with either agonistic or antagonistic TNFR2 targeting reagents depends on the individual context, such as immune status, tumor type and more factors.

## Conclusion and Outlook

Tumor necrosis factor blockers have demonstrated their clinical effectiveness, are successfully used to treat autoimmune diseases and are under the top-selling biologics world-wide. However, despite this success the development of serious side-effects and the failure of clinical trials in specific indications such as heart disease and MS revealed the limitations of anti-TNF therapy. Research of the last two decades has established that TNF mediates inflammation and tissue degeneration via TNFR1 signaling and immunomodulation and tissue regeneration via TNFR2. Accordingly, a novel class of drugs that selectively target TNF signaling at the level of the ligand or receptor has emerged. As outlined in this review, selective blocking of sTNF/TNFR1 signaling, which will preserve functional tmTNF/TNFR2 signaling, seems to be sufficient to interfere with pathological TNF signaling. In contrast to global TNF blockers that neutralize sTNF and tmTNF, this class of therapeutics may induce less severe side-effects and may be therapeutic for other diseases such as MS or neurodegenerative diseases, where complete TNF inhibition is contraindicative. Indeed, preclinical evaluation of DN-TNF muteins and TNFR1 antagonists was promising and often superior to conventional anti-TNF therapeutics. Similar, TNFR2 agonists were developed and first pre-clinical evaluation using prototype molecules was successful. However, development of completely human clinical grade products will be necessary to succeed into clinical trials. Ultimately, combination therapies, using sTNF/TNFR1 antagonists together with TNFR2 agonists, may rebalance pathologically deregulated TNF signaling and induce tissue repair and might be a novel superior therapeutic concept to treat a multitude of inflammatory and degenerative diseases.

## Author Contributions

RF and RK wrote the review and generated the figures. KP reviewed and revised the manuscript.

## Conflict of Interest

RK and KP are named inventors on patent applications covering TNFR1 specific antagonists. RF, RK, and KP are named inventors on patent applications covering the TNFR2 agonist technology.
